# Supplementary material for: Knocking out histidine ammonia-lyase by using CRISPR-Cas9 abolishes histidine role in the bioenergetics and the life cycle of Trypanosoma cruzi
Source: Microb Cell. 2025 Jun 25;12:157–72. doi: 10.15698/mic2025.06.853 (PMC12203737; doi:10.15698/mic2025.06.853)
Supplement: Supplementary file 1 [file mic-12-157-s01.pdf]

Knocking out histidine ammonia-lyase by using CRISPR-Cas9 abolishes histidine role in the bioenergetics and the life cycle of *Trypanosoma cruzi*

Janaína de Freitas Nascimento[1], María Julia Barisón[1], Gabriela Torres Montanaro[1], Letícia Marchese[1], Rodolpho Ornitz Oliveira Souza[1], Letícia Sophia Silva[2], Alessandra Aparecida Guarnieri[2] and Ariel Mariano Silber[1,*]

[1] Laboratory of Biochemistry of Tryps - LaBTryps, Instituto de Ciências Biomédicas, Universidade de São Paulo, São Paulo, Brazil. [2] Vector Behaviour and Pathogen Interaction Group, Instituto René Rachou, Belo Horizonte, MG, Brazil.

* Corresponding Author:
Ariel Mariano Silber, Laboratory of Biochemistry of Tryps - LaBTryps, Instituto de Ciências Biomédicas, Universidade de São Paulo. Av. Lineu Prestes 1374, (05508-000) Cidade Universitária São Paulo – SP, Brazil; Tel: +55-11-3091-7752; E-mail: asilber@usp.br

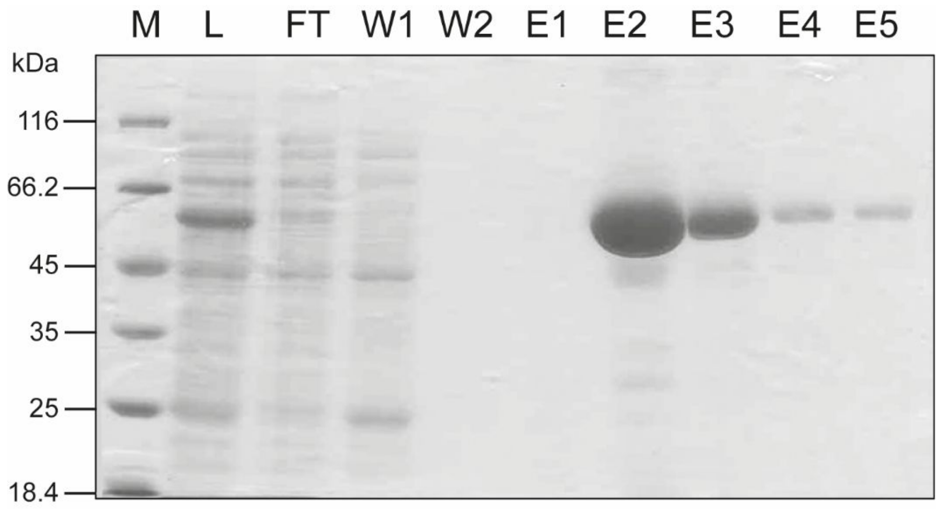

Supplemental Figure S1: Expression of recombinant *Tc*HAL. Fractions from recombinant *Tc*HAL affinity chromatography were resolved on SDS-PAGE. M: molecular mass marker; L: clarified lysate; FT: flow-through; W1 and W2: washes using 60 mM imidazol, E1-5: elutions using 500 mM imidazol.

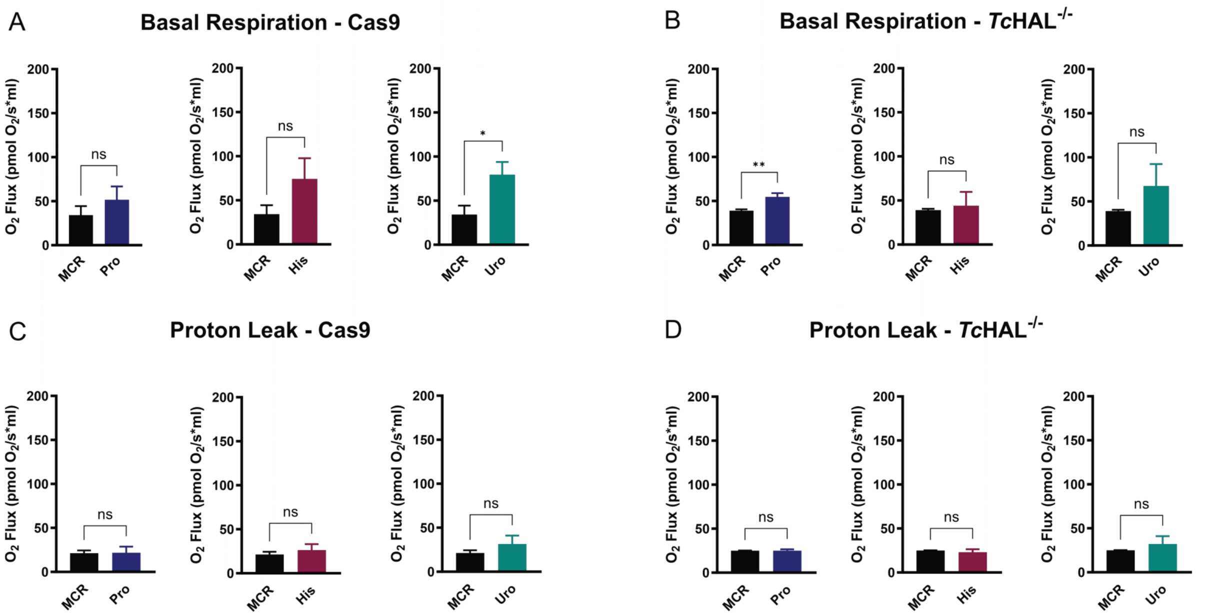

Supplemental Figure S2: Knockout of *Tc*HAL does not affect basal respiration and proton leak. Quantification of Basal Respiration in **(A)** Cas9 and **(B)** *Tc*HAL$^{-/-}$ and Proton Leak in **(C)** Cas9 and **(D)** *Tc*HAL$^{-/-}$ shows no difference when parasites are recovered in proline (positive control), histidine or urocanate, when compared to cells recovered only in MCR buffer. Error bars represent the standard deviation of experiments performed with three independent clones for each strain (Cas9 and *Tc*HAL$^{-/-}$) for each condition. Statistical analysis was performed using unpaired t-test, using MCR as control (ns = statistically non-significant difference, * $p \leq 0.05$).

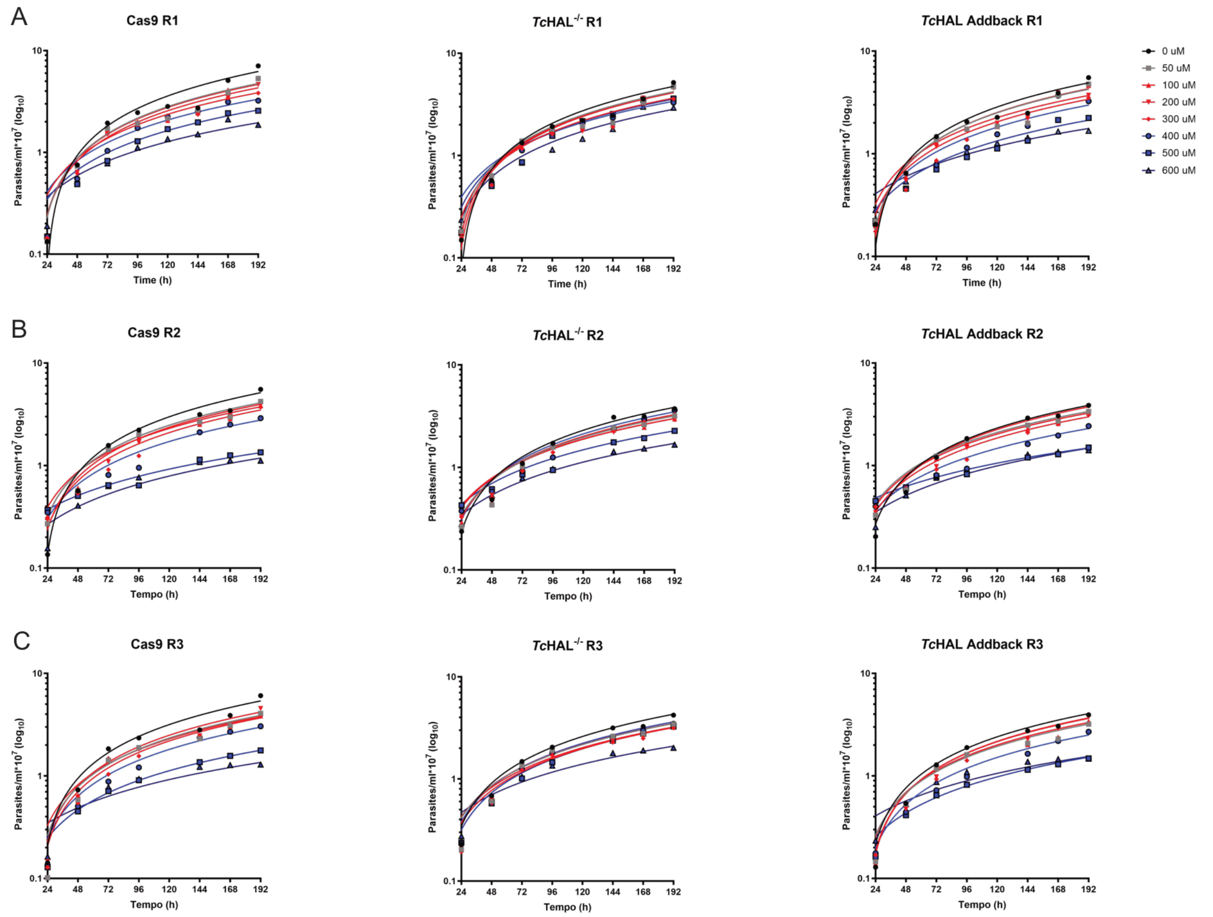

Supplemental Figure S3: Epimastigotes of the *Tc*HAL$^{-/-}$ cell line are less sensitive to nickel. The proliferation of epimastigotes was monitored by spectrophotometric reading of optical density (620 nm) every 24 hours for 192 hours in the presence of indicated concentrations of $NiSO_4$. Graphs show the results of three biological replicates **(A)** R1, **(B)** R2 and **(C)** R3 performed for Cas9, *Tc*HAL$^{-/-}$ and *Tc*HAL add-back epimastigotes, adjusted to the exponential growth model. Values of the growth rate constant (k) and doubling times with α=0.05 were estimated and are shown in Supplemental Table S2.

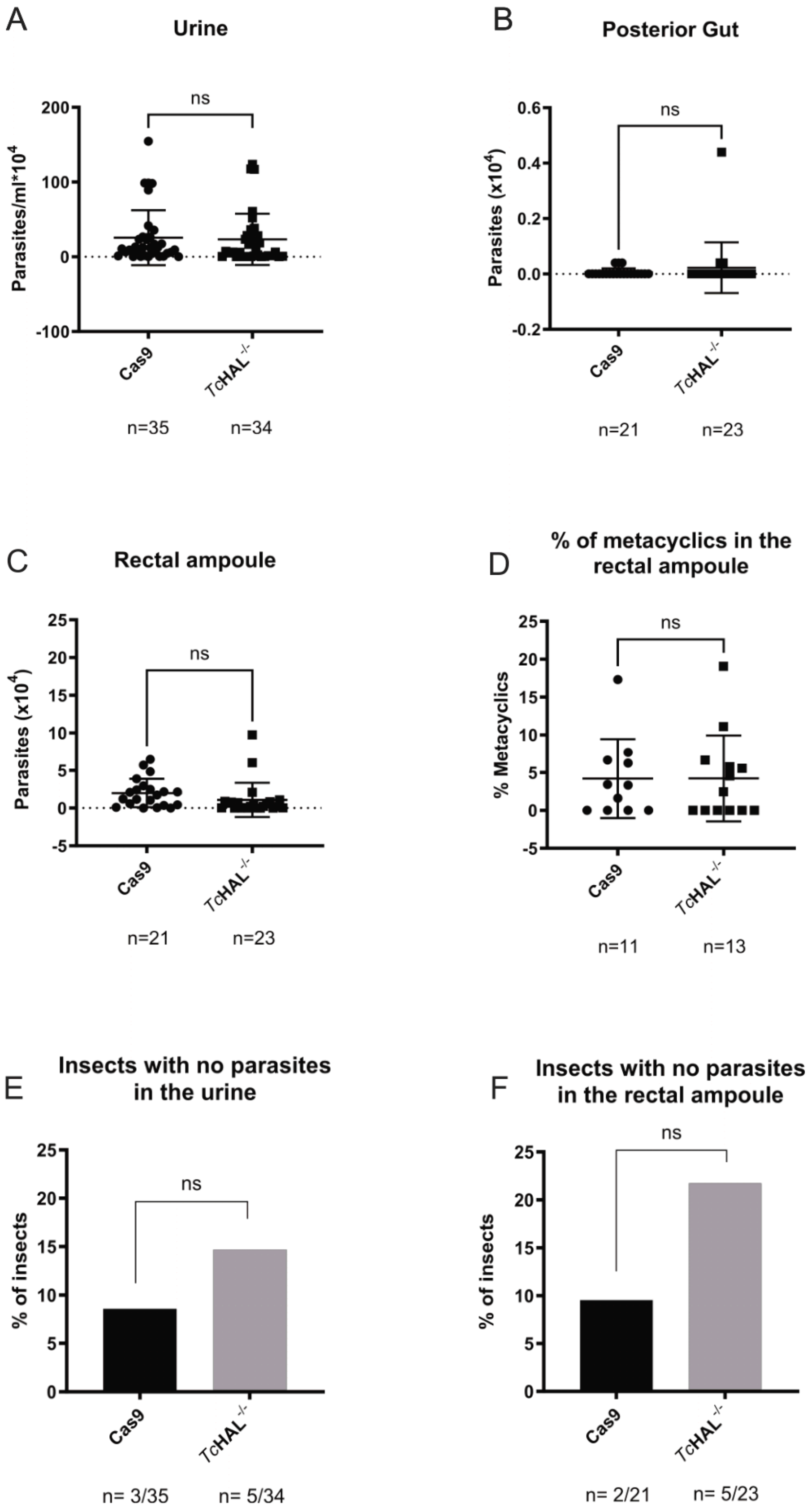

Supplemental Figure S4: Knockout of *Tc*HAL does not affect the parasites' infection capability in the insect vector. Groups of insects were infected by feeding with epimastigotes of the Cas9 or the *Tc*HAL$^{-/-}$ cell lines. Twenty days after infection, the triatomines were fed again, and urine was collected from the insects for parasite counting (A). The next day, the insects were dissected, and the presence and stage of the parasites in the posterior gut (B) and rectal ampoule (C and D) were assessed. Statistical analysis was performed using unpaired t-tests, using Cas9 as control (ns = statistically non-significant difference). The percentage of insects with urine (E) and rectal ampoule (F) negative for parasite presence was also evaluated. Note the difference in the y-axis of the graphs. Statistical analysis was performed using Z test for proportions (α=0.05, ns = statistically non-significant difference).
